# Posturographic characteristics of the standing posture and the effects of the treatment of obesity on obese young women

**DOI:** 10.1371/journal.pone.0220962

**Published:** 2019-09-04

**Authors:** Joanna Magdalena Cieślińska-Świder, Janusz Wiesław Błaszczyk

**Affiliations:** 1 The Jerzy Kukuczka Academy of Physical Education, Department of Physiotherapy of the Nervous System and the Musculoskeletal System, Katowice, Poland; 2 The Jerzy Kukuczka Academy of Physical Education, Department of Human Motor Behavior, Katowice, Poland; Universidade Estadual Paulista Julio de Mesquita Filho, BRAZIL

## Abstract

To determine the impact of body weight on quiet standing postural sway characteristics in young women, this research compared spontaneous oscillations of the center of foot pressure (COP) between 32 obese (BMI: 36.4 ± 5.2 kg/m^2^), and 26 normal-weight (BMI: 21.4 ± 1.5 kg/m^2^) women and assessed the influence of obesity treatment and body weight reduction on postural sway. Trajectories of the COP were assessed while the subjects were standing quietly with eyes open (EO) and closed (EC). Both in the sagittal (AP) and frontal (ML) planes the sway range, average velocity, and maximal velocity of COP were calculated. Moreover, the total average and maximal velocities were computed. In the obese group, the tests were performed twice–before and after the obesity treatment. A greater (18% in EC) AP sway range and a substantial reduction of ML sway (25% in EO, 22% in EC) were observed in the obese women. The total COP velocities (average and maximal) were decreased in obese women (20% and 20% in EO) as well as the velocities in the frontal plane (EO: 33%, 41%; EC: 34%, 40%). Body weight reduction resulted in significant changes in postural sway. The following parameters increased: ML sway range (28% in EO), average (20% in EO, 16% in EC) and maximal ML (20% in EO) velocities. The results indicate that young obese women in the habitual standing position are characterized by the destabilizing influence of mass in the sagittal plane only in the absence of a visual control. This effect is dominated by the stabilizing mass effect in the frontal plane, which affects overall postural stability when standing. The reduction of body mass enables a decrease in ML static stability, likely due to natural changes in the base of support while standing.

## Introduction

Human posture is characterized by the vertical orientation of the body in relation to a small base of support [1]. This fact presents a fundamental challenge for balance control. Balance is commonly modeled as a process of active control of an inverted pendulum with the pivot point located at the ankle joints [2,3]. The central nervous system controls the pendulum, generating force in the ankle stabilizing muscles [3,4,5]. This multimodal (integrating visual, vestibular, and proprioceptive inputs) and redundant control, due to numerous substantial delays and nonlinearities, causes the inverted pendulum to perform tiny chaotic COM (center of mass) oscillations known as postural sway [1,3,6,7]. In contemporary laboratories and clinics, various measures of postural sway are utilized to assess the quality of balance control [1,7]. In static posturography, COM oscillations are represented by the center of foot pressure (COP) displacements [8,9].

Generally, postural stability can be viewed as its ability to persist and remain qualitatively unchanged in response to interferences or fluctuations (including postural sway) in the control [1]. There are a number of factors which determine both postural sway and postural stability, but the body’s anthropometry (height and weight) seems to be one of the most significant factors [10,11]. As the first to do so, Fregly et al. (1968) recognized the relationship between the body’s anthropometry and balance control [12]. Abdominal circumference, endomorphy, and body weight were identified as the most important factors affecting the performance of military recruits in postural tests [12]. Consequently, it was suggested that individuals with excessive body weight may experience balance impairments due to an altered COM position [13,14,15,16].

Obesity is accumulated excess fat within the body causing many health problems such as diabetes, high blood pressure, chronic heart diseases, dyslipidemia, stroke, cancers, osteoarthritis, and respiratory problems [17,18]. Obesity requires absolute treatment, including fat mass reduction [19] because of medical indications [18] and also to reduce the risk of falling in the obese [20] by increasing mobility.

In the literature, it is commonly claimed that postural stability in obese subjects decreases as result of increased body sway [12]. Consequently, most posturographic studies in obese subjects documented increased postural sway [21,22,23,24,25,26,27]. In contrast to the aforementioned studies, reduced postural sway was observed in obese women, especially in relation to the frontal plane [28,29,30]. Undoubtedly, anthropometric differences [9,31], in this degree and location of adipose tissue [32], could have specific effect with regards to body mass on postural control in obese men and women, due to a different COM location. Therefore, Menegoni et al. (2009) observed that body mass correlated with postural instability in the antero-posterior direction in both genders, but in the medio-lateral direction only in obese men [25]. In the face of this discovery, we are interested in the directional postural control analysis in obese women for a better understanding of this phenomenon.

Previous studies on postural control mainly concerned middle-aged [25,28,29], postmenopausal [33] or older [23,26,30] obese women. According to our knowledge, postural stability in young women has not been studied thus far. We are particularly interested in young women because they are characterized by the gynoid type of obesity, which results in a lowering of the COM position since the adipose tissue is typically found around the hips and thighs [34], and therefore they may present a different posturographic characteristic. When we were examining young women we also wanted to avoid the influence of diseases associated with long-term obesity on postural control such as, for example, diabetic neuropathy in diabetes [35].

Therefore, the purpose of the present study was to assess the impact of body weight on postural stability during an upright stance in obese young women. For this purpose, we wanted to compare the postural sway characteristics of obese and normal-weight women. We also wanted to investigate the influence of obesity treatment, especially body weight reduction on postural stability, as weight reduction changes the body’s geometry. Based on our previous results, we put forward the hypothesis that a greater weight in this population may improve static postural stability. A reduction in weight may reduce static postural stability and increase mobility in the obese, which will help them control their daily activities, including those associated with the risk of falling.

## Materials

A total of 32 obese women (group O) were tested in this study. The mean age of the subjects was 35.9 ± 9.8 years, and the mean of the body mass index (BMI) of the study group was 36.4 ± 5.2 kg/m^2^. The control group (group C) consisted of 26 age-matched women, with the mean age of the group being 36.1 ± 11.4 years. Control subjects exhibited normal body weight (58 ± 5.2 kg) based on BMI (21.4 ± 1.5 kg/m^2^).

**Fig 1:**
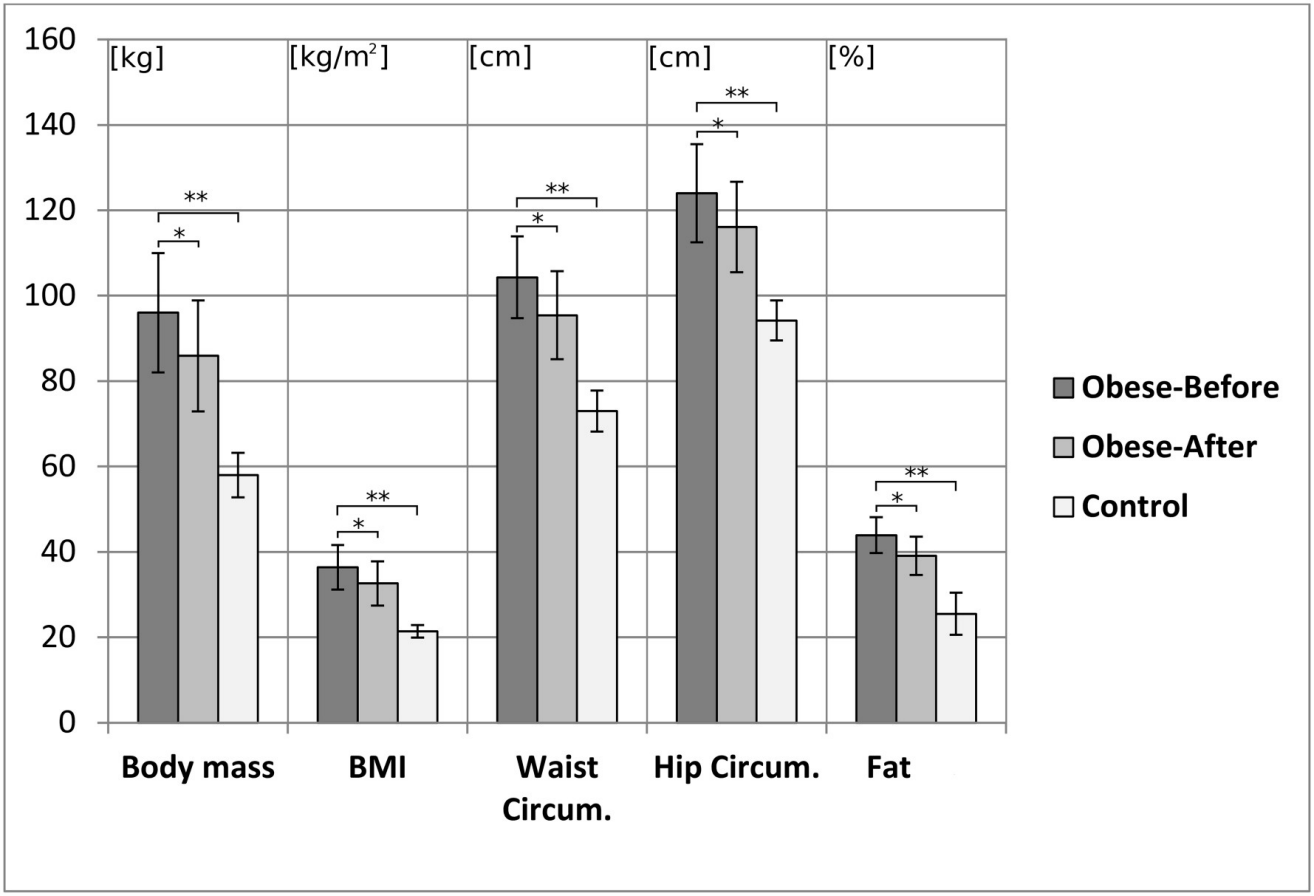
Group characteristics, difference between Obese-Before therapy vs. Control **p<0.001 and Obese-Before therapy vs. Obese-After therapy * p<0.001.

Body height did not differentiate groups (O: 162.6 ± 5.6 cm vs C: 164.4 ± 5.2 cm, p = 0.1917). The characteristics of the groups are presented in Fig 1. All subjects had to be free from severe musculoskeletal disorders, especially a deformity or injury of the lower extremity and vertebral column, uncorrected vision problems, balance disorders, cardiovascular disorders, diabetes, and mental disorders, and were not pregnant at the time of testing (these were exclusion factors from the study). Obese patients were also asked about the cause and duration of their obesity as well as the accompanying diseases. All participants gave their informed consent to participate in the study, which was approved by the Senate Ethics Committee of the Katowice Academy of Physical Education. Anthropometric and posturographic tests were performed twice in the study group, before and after the weight loss program. The weight loss program consisted of a reduced diet (1200 kcal per day) and physical activity in the form of a) increased daily physical activity (walking, using stairs, manual car washing, etc.), and b) additional exercise (swimming, cycling, group exercises, etc.). The subjects had to perform physical activity 3–7 times per week for 30–60 minutes and should keep within 60–70% of their maximum Heart Ratio during exercises [36,37]. In the weight loss program, 120 obese women participated and were qualified by their physician internist for the treatment of obesity. During the treatment, obese subjects had to come to the Center of Metabolic Diseases and Treatment of Obesity every two weeks for a control-consultation session with a doctor, nutritionist, psychologist, and a physiotherapist. The program was implemented for one year. The experiment assumed observations before and after 3 months of therapy. Criteria for inclusion in the study were: age 45 years and under, and a body mass reduction equal to or greater than 5% of the initial mass. Control subjects (C) were tested only once.

## Methods

### Anthropometric measurements

The Tanita weighing platform and body composition analyzer (TBF 300P type, Tanita Corporation, Tokyo, Japan) was used for body mass (kg) and body fat content (%, based on the bioelectrical impedance analysis) measure. The body mass index (the ratio of an individual’s body mass in kg and her height in m squared) was calculated for each subject. Additionally, waist and hip circumference was measured. The hip circumference was measured around the widest portion of the buttocks. The waist circumference was measured at the midpoint between the lower edge of the costal arch and the top of the iliac crest, with the tape parallel to the floor and perpendicular to the long axis of the body. All measurements were taken using stretch-resistant tailor’s tape, according to the WHO’s data gathering protocol (2008, [38]).

### Stabilometric tests

The Kistler 9281C force platform (Kistler Group, Switzerland) was used to record the ground reaction forces and the moments around the sagittal and frontal axis based on which the center of foot pressure (COP) was calculated (BioWare 2.0 software). Signals from the sensors were sampled at 100 Hz by a 16-bit analog-to-digital converter. The COP signals were filtered then with a low-pass filter (Butterworth 4th order, type I) at cutoff frequency of 7 Hz to reduce the measurement noise [1,39]. A custom-developed software was used to compute the COP parameters [40], and maximal COP velocity as additional parameter [32]. The formulas by which the variables were calculated were implemented in C++. Only tools available under free licenses were used. The calculated parameters and formulas by which they were calculated are presented below:

- the sway range in the sagittal plane (Range AP):
  $$\left(max({AP}_{i})-min({AP}_{i}))\;for\;all\;i\in \langle 50,n\right\rangle$$
- the sway range in the frontal plane (Range ML):
  $$\left(max({ML}_{i})-min({ML}_{i}))\;for\;all\;i\in \langle 50,n\right\rangle$$
- the average velocity in the sagittal plane (V_avg_ AP):
  $$\frac{1}{n}\sum_{i=50}^{n}\frac{\left|{AP}_{i}-{AP}_{i-1}\right|}{\Delta t}$$
- the average velocity in the frontal plane (V_avg_ ML):
  $$\frac{1}{n}\sum_{i=50}^{n}\frac{\left|{ML}_{i}-{ML}_{i-1}\right|}{\Delta t}$$
- the maximal velocity in the sagittal plane (V_max_ AP):
  $$max(\frac{{AP}_{i}-{AP}_{i-1}}{\Delta t})\;for\;all\;i\in \langle 50,n\rangle$$
- the maximal velocity in the frontal plane (V_avg_ ML):
  $$max(\frac{{ML}_{i}-{ML}_{i-1}}{\Delta t})\;for\;all\;i\in \langle 50,n\rangle$$
- the total average velocity of the COP displacement (V_avg_ TOT):
  $$\frac{1}{n}\sum_{i=50}^{n}\frac{\sqrt{{\left({AP}_{i}-{AP}_{i-1}\right)}^{2}+{\left({ML}_{i}-{ML}_{i-1}\right)}^{2}}}{\Delta t}$$
- the maximal velocity of the COP displacement (V_max_ TOT):
  $$max(\frac{\sqrt{{\left({AP}_{i}-{AP}_{i-1}\right)}^{2}+{\left({ML}_{i}-{ML}_{i-1}\right)}^{2}}}{\Delta t})\;for\;all\;i\in \langle 50,n\rangle$$

i sample number

AP_i_ COP location in the sagittal plane at time i

ML_i_ COP location in the frontal plane at time i

n number of samples

Δt sampling period, 0.01s

All subjects participated in three 30-second trials with their eyes open (EO) and three 30-second trials with eyes closed (EC). The mean values obtained from all the trials, for each visual condition (EO and EC), were used in the further analysis. The first 0.5 seconds recorded during each trial was excluded from further analysis.

### Experimental procedure

Before each trial, the task was explained clearly so that each subject knew how to perform it. During posturographic tests each subject was asked to stand barefoot and at a comfortable stance on the platform. All subjects chose an open stance with feet apart and slightly turned out, keeping hands along the torso, and looking straight ahead at the wall (or eyes closed), for sound signal subject had the task “stand as still as possible”. Particular attention was paid to keeping the distance between feet shorter than the shoulder width [40]. To ensure foot position remained constant, tracings of foot placements were made. Subjects were required to remain on their traced positions for all of the trials. The 30s trials were separated with short resting breaks to avoid fatigue or boredom.

### Statistical analysis

The parameters for each group were quantitatively analyzed using the arithmetic mean value and standard deviation. To verify the normal distribution of the analyzed data, the W Shapiro-Wilk test was used. The Pearson’s correlation between body weight, body mass index, body fat percentage, waist and hip circumference, and parameters of postural stability was assessed. Statistically significant differences between the study and the control groups were determined using the non-parametric U Mann-Whitney test for independent variables. The non-parametric Wilcoxon signed rank sum test for dependent variables was used to detect statistically significant differences between the initial and final test in the group of obese women. All results were considered to be significant at the p<0.05 level. For all analyses, Statsoft Statistica 9 software was used.

## Results

### Postural sway characteristics in obese women

Statistical analysis revealed significant differences between obese women and the control group in all anthropometric indices (Fig 1). Further analysis revealed significant differences between groups in postural sway characteristics (Table 1).

**Table 1:** Main center of foot pressure (CP) parameters describing spontaneous sway in obese and control groups (O vs. C) while standing for 29.5s with eyes open and eyes closed; ns = not statistically significant.

Eyes	Group	SR AP (mm)	SR ML (mm)	V_avg_TOT (mm/s)	V_avg_AP (mm/s)	V_avg_ML (mm/s)	V_max_TOT (mm/s)	V_max_AP (mm/s)	V_max_ML (mm/s)
OPEN	O	17.2±5.1	11.8±5.0	8.1±2.3	6.1±1.9	5.0±1.4	32.6±10.9	27.0±10.2	19.5±6.9
	C	17.5±5.8	15.8±5.2	10.1±2.8	6.0±1.5	7.5±2.4	40.6±13.1	28.3±7.3	33.1±14.6
	p<	ns	0.01	0.01	ns	0.00001	0.00001	ns	0.00001
CLOSED	O	24.6±7.2	14.8±6.6	11.9±3.7	9.4±3.3	6.1±1.9	54.7±23.1	46.0±21.2	27.0±14.6
	C	20.8±7.1	18.9±6.8	13.4±4.2	8.4±2.3	9.3±3.6	62.1±22.2	43.5±15.7	45.3±25.5
	p<	0.05	0.05	ns	ns	0.001	ns	ns	0.001

SR—sway range, V_avg_—average velocity of the COP displacement, Vmax—maximal velocity of the COP displacement, TOT—total, AP—in sagittal plane, ML—in frontal plane.

In EO conditions in the obese group, the sway range in the frontal plane was significantly reduced (25%) in comparison with the normal-weight subjects (Table 1). It was also observed that the total velocities (average and maximum) were decreased in obese woman (20% and 20%), especially in velocities in the frontal plane (33% and 41%) (Table 1).

While standing with EC the sway range in the antero-posterior direction was significantly greater (18%) in the obese women compared to the women with a normal body weight, and the sway range in the frontal plane was significantly reduced in comparison with the normal-weight subjects (22%). Additionally, the average and maximum sway velocities in the frontal plane were reduced (34% and 40%) in the obese group in eye closed conditions (Table 1).

### Correlations

Significant correlations between body indicators and posturography variables for all visual conditions were found. Detailed results are shown in Table 2.

**Table 2:** The Person’s correlations between the anthropometric and COP variables in obese and control subjects (n = 58), during quiet standing with eyes open and eyes closed conditions. The table shows the correlation coefficient (r). All shaded cells are showing significant correlation with p<0.05.

Eyes		SR AP	SR ML	V_avg_TOT	V_avg_AP	V_avg_ML	V_max_TOT	V_max_AP	V_max_ML
OPEN	Body mass	0.07	-0.29	-0.23	0.16	-0.49	-0.15	0.05	-0.42
	BMI	0.04	-0.33	-0.26	0.13	-0.49	-0.19	0.03	-0.47
	Fat %	0.07	-0.35	-0.28	0.11	-0.53	-0.25	0.00	-0.49
	Waist	0.03	-0.39	-0.28	0.11	-0.51	-0.24	0.03	-0.50
	Hip	0.09	-0.31	-0.18	0.22	-0.46	-0.12	0.11	-0.42
CLOSED	Body mass	0.37	-0.15	-0.09	0.28	-0.40	-0.09	0.08	-0.36
	BMI	0.32	-0.21	-0.11	0.30	-0.45	-0.10	0.08	-0.38
	Fat %	0.26	-0.22	-0.13	0.26	-0.47	-0.15	0.09	-0.37
	Waist	0.25	-0.29	-0.11	0.31	-0.46	-0.10	0.11	-0.39
	Hip	0.35	-0.15	-0.03	0.36	-0.39	-0.04	0.16	-0.32

SR—sway range, V_avg_—average velocity of the COP displacement, Vmax—maximal velocity of the COP displacement, TOT—total, AP—in sagittal plane, ML—in frontal plane.

### Impact of obesity treatment on postural sway

As a result of obesity treatment, there were changes in the measured anthropometric parameters. All anthropometric features, including body weight and BMI, fat tissue, and waist and hip circumferences, were significantly reduced (Fig 1). The observed changes resulted in the modification of postural sway characteristics (Table 3).

**Table 3:** Effects of body weight reduction in obese women on postural stability. Main center of foot pressure parameters describing spontaneous sway in the Obese-Before and Obese-After groups (Before vs. After) while standing quiet for 29.5s with eyes open and eyes closed; ns = not statistically significant.

Eyes	Obese Group	SR AP (mm)	SR ML (mm)	V_avg_TOT (mm/s)	V_avg_AP (mm/s)	V_avg_ML (mm/s)	V_max_TOT (mm/s)	V_max_AP (mm/s)	V_max_ML (mm/s)
OPEN	Before	17.2±5.0	11.8±5.0	8.1±2.3	6.1±1.9	5.0±1.4	32.6±10.9	27.0±10.2	19.5±6.9
	After	18.9±8.5	15.1±5.1	8.8±2.1	6.0±1.6	6.0±1.6	33.9±10.1	27.6±8.6	23.4±8.8
	p<	ns	0.01	0.08	ns	0.05	ns	ns	0.01
CLOSED	Before	24.6±7.2	14.8±6.6	12.0±3.7	9.4±3.3	6.1±1.9	54.7±23.1	46.0±21.2	27.0±14.6
	After	24.4±8.0	16.1±6.1	11.9±3.8	8.7±3.1	7.1±2.7	52.6±19.9	42.7±14.9	30.8±15.8
	p<	ns	ns	ns	ns	0.05	ns	ns	ns

SR—sway range, V_avg_—average velocity of the COP displacement, Vmax—maximal velocity of the COP displacement, TOT—total, AP—in sagittal plane, ML—in frontal plane.

## Discussion

### Body sway characteristics during a static upright posture

Our present results confirmed that the sway characteristics in obese women differed significantly compared with their normal-weight counterparts. Interesting insights are provided by the directional parameter analysis in our study. Generally we found opposite effects of increased body mass on balance control in the sagittal and frontal planes in young obese women, what impacted overall postural balance (total value from both directions).

The COP velocity parameters are often considered to represent the overall amount of activity to maintain stability [22], while the parameters related to the magnitude of COP displacements (Ranges) are often claimed to be related to the effectiveness of the postural control system [41]. Higher values of the COP velocity [42,43] and ranges [44] are commonly interpreted as an impairment of balance control. In fact, good static balance control is characterized by lower values [45].

#### Sagittal plane

Our experiment showed no significant differences in velocities in the antero-posterior (AP) direction (V_avg_AP, V_max_AP) in obese and normal weight women with and without vision control; however, a clear trend was confirmed, supported by recorded significant positive correlations of the average velocity in AP (V_avg_AP) with anthropometric parameters including mass (r = 0.28–0.36, see Table 2), observed with no visual control. At the same time, Range AP significantly increased in the sagittal plane in the eyes closed condition in obese women when compared to normal-weight women.

The results of other studies in obese women have shown a greater destabilization impact for body weight on postural control in the sagittal plane; the higher COP velocities [25,29,30] and larger AP ranges [26] were noted with and without a vision control. In addition, in Menegoni et al. (2009, [25]) the same effects were demonstrated by examining women with a visual control only. Conversely, Błaszczyk et al. (2009) did not find differences between obese and normal weight women in AP postural control [29].

From a biomechanical point of view, differences between the obese and the normal-weight could be explained by adopting the hypothesis proposed by Winter et al. (1996) that the control postural system can be described with an inverse pendulum model [2]. The stability of human upright posture is controlled in the sagittal plane by the ankle joint stabilizers [2,3]. The increased body mass (the higher COM location in relation to the ankle joint) in obese individuals causes an increase of torque at the ankle level and consequently an increased demand on muscle strength and activity to maintain the COP within the base of support [21,46]. Because obese individuals have lower relative muscle strength (e.g. ankle joint stabilizing muscles related to body weight) than those with a normal body mass [47,48,49], they have a reduced capacity to control sways.

The destabilization effect of mass on AP direction in obese women in our study was involved in the lack of visual control. The constraint of the afferent sensory information (tele-receptors) causes an impairment of postural stability [50]. Trials without visual feedback usually supplement the postural study because they allow for the investigation of additional factors influencing postural control [9]. The observation of no differences between groups in trials with a visual control in our study may support the notion that the decline in postural stability in the examined obese population is at least partially compensated by increased visual feedback [51,52].

#### Frontal plane

The presented work indicates the greatest effect of increased body weight on posture control in the medio-lateral (ML) plane. All velocity parameters in the ML plane (V_avg_ML, V_max_ML) were significantly reduced in obese women in comparison to normal-weight women in both visual conditions. At the same time, Range ML revealed a substantial decrease in vision and non-vision conditions in obese women when compared to normal-weight women. The results show the stabilizing effect of body mass on postural control in the frontal plane. These results are confirmed by moderate negative correlations between the anthropometric variables and velocities. The strongest effect was observed for average ML velocity in the eyes open condition for mass (r = -0.49), body fat percentage (r = -0.49), as well as waist (r = -0.51) and hip (r = -0.46) circumferences. These observations are consistent with earlier results [28] and other studies [29,30]. However, Menegoni [25] did not notice significant differences between obese and normal-weight women in ML COP displacements.

Postural control in the frontal plane (in the inverted pendulum model) mostly depends on ‘‘hip level control” rather than ankle muscle control [2], as well as on the width of the support area [7,53]. The observed changes in our study result not only from an increased body weight that can be considered as a relative decrease of muscular forces (as discussed above), but also from the natural modification of the base of support in obese women when standing naturally. In fact, ankle joint mobility in the frontal plane is reduced when standing widely [54,55]. The width of the base of support was not measured in the presented experiment; however, the results from the literature [56,57] show that obese individuals adopt a wider stance, which is also a kind of more effective strategy to compensate for increased body mass. The modification of the width while standing was observed in women with increasing body mass during pregnancy [58].

In the present investigation, all participants stood barefoot with a natural foot position on the force plate. We agree with the concept that the forced position of the feet would be interfering with the equilibrium of the body and therefore would impact the control of posture stability [40]. In terms of energy, the maintenance of a comfortable body posture is also connected with the search for the optimal (minimal) energy expenditure required to hold an upright body position [59]. Moreover, the practical aspect enforces the natural feet position during standing position. People with obesity frequently have additional health problems such as faulty posture, in terms of valgus knees and ankles, which prevent the feet from being kept together. In addition, the enlarged circumference of the thighs in obese young women (gynoidal type of obesity) leads to difficulties with the standardization of the feet position during investigation.

#### Differences in the results

In reference to the discussion with the results of other authors who examined obese women, we wish to present some of the possible causes of the differences in the results. The first of these is the impact of physical activity on postural control. In our study, we did not study muscle strength or level of physical activity; however, our subjects were young, fit, and physically active on a daily basis. It was found that exercises repeated on a daily and weekly basis improve postural control [60,61] and can generate functional and structural adaptation in the neuromuscular system [61]. It is also necessary to take into account customary factors preferred by young women, such as walking in high heels on a daily basis. Another, in our opinion important, reason for the discrepancy between our results and those of others is the biomechanical effect of fat distribution on COM positioning. The authors’ current and earlier [28] research concerns women who mostly present a gynoid type of fatness. It should be noted that women in other works were older than those in our experiment. Due to estrogen deficiency associate with aging, the distribution of fat tissue in women changes–women’s obesity becomes men’s obesity [62]. The influence of adipose tissue localization on postural control in obese women has been proven [32,33]. A larger range in the AP direction and the higher value of the maximum average velocity has been shown in the obese with body mass was highly located—in android type [33]. It should be also noted that the distribution of additional mass, in addition to the impact of the COM position, may also affect the size of the support surface [58].

In addition, other physiological age-related [44] factors may influence the observed differences. The different results quoted in other studies [26,27] may also arise from the different measurement conditions during testing (standing with their feet together).

### Postural characteristic in obese women after obesity treatment

As a result of obesity treatment, body anthropometric factors were changed (see Fig 1). Consequently, when considering body mass reduction in the obese, the characteristics of postural stability in static conditions changed and moved closer to the characteristics of normal-weight women (see Table 3). Due to the reduction in body weight, we did not notice the influence of the therapy on the COP total velocities during standing, only trend was observed.

Based on the analysis of directional changes in the COP parameters, we recorded their increase (V_avg_ML, V_max_ML, Range ML) as a result of body weight therapy. We consider that the observed changes are associated with natural changes in base of support after body weight reduction. A similar effect was observed in women in advanced pregnancy and after delivery [58]. In addition, we have not studied this aspect accurately in this study, but reduced circumferences in the thighs after therapy may have influenced the change in the width of the support. In our previous study [63], we found significant changes in thigh circumferences as a result of a weight loss exercise program.

### Limitation

The control of postural stability is complex. Multiple factors may influence the results. The biggest limitation of the work was the lack of measurement of the foot position before and after the intervention; thigh circumference should also be measured in the context of natural changes in the support width in response to reduced mass.

### Conclusion

In conclusion, young obese women in a natural standing position are characterized by the destabilizing influence of mass in the sagittal plane only in the absence of a visual control. This effect is dominated by the stabilizing mass effect in the frontal plane, which affects overall postural stability when standing and shows that obese women in the natural standing position are more stable than those with a normal body weight. The reduction of body mass enables a decrease in ML static stability, likely due to natural changes in the base of support when standing. This effect may have an influence on the increase of mobility in obese and the better dynamic control required for any motor activity or a response to perturbation or danger. Further tests are necessary to determine the issues related to dynamic stability.

## Supporting information

S1 Dataset Study dataset. (XLSX)
